# Skull base metastases from a malignant solitary fibrous tumor of the liver. A case report and literature review

**DOI:** 10.1186/1746-1596-6-127

**Published:** 2011-12-22

**Authors:** Lu Peng, Yang Liu, Yongbiao Ai, Zhisu Liu, Yueming He, Quanyan Liu

**Affiliations:** 1 Department of General Surgery, Research Center of Digestive Diseases, ZhongNan Hospital, Wuhan University, Wuhan 430071, P.R. China

**Keywords:** Solitary fibrous tumor, Liver

## Abstract

Solitary fibrous tumors (SFTs) of the liver are rarely described; only 38 cases have been reported in literature, most of which have shown benign clinical characteristics, and only 3 of these cases exhibited malignant variants. In this study, we present a 24-year-old woman with a 1-month history of a rapidly enlarging abdominal mass and a CT showing an exophytic heterogeneous liver mass with a firm parietal bone mass. The patient underwent a transcatheter arterial chemoembolization (TACE) before operation, and an extended right hepatectomy and craniectomy with a negative margin was performed under general anesthesia. The masses showed histological features of oval spindle cells haphazardly arranged in the classic short-storiform or so-called patternless pattern of solitary fibrous tumors. The tumor cells showed positive immunohistochemical reactions to CD34 and bcl-2. The tumor recurred in the residual liver 2 months after operation, metastatic osteoblastic lesions in the thoracic and lumbar vertebrae were identified 3 months after the operation, and lumbar vertebrae metastasis 7 months after operation paralyzed the patient. The patient underwent percutaneous ethanol injection therapy (PEI) and chemotherapy, but the patient died because of the uncontrolled tumor 16 months after the initial operation. To our knowledge, this is the first case of malignant solitary fibrous liver tumors with skeletal metastasis.

## Introduction

Solitary fibrous tumors (SFTs) are a rare soft-tissue neoplasm that was initially described most commonly in the pleural cavity[1]. In the past decade, SFTs have been discovered at many body sites, including the liver (mostly the right lobe), orbits, superior respiratory tract, abdomen, breast and soft tissue [2-4]. SFTs are spindle cell neoplasms. The histogenesis of SFT has been a matter of contention and still has not been definitively discerned, but a mesenchymal origin is favored over a mesothelial origin[5].

More than 80% of SFTs are benign, asymptomatic and slow-growing tumors, but malignant and symptomatic forms, most often in the pleura, have been described [6,7]. Clinical and radiological findings are not specific and cannot exclude malignancy. Preoperative cytology may be inconclusive or misleading. Notably, all SFTs that recurred or metastasized after resection in some larger series were ≥ 10 cm [8,9]. In addition, several authors describe a histologically malignant component with increased cellularity and mitoses in otherwise benign-appearing SFTs, as a frequent occurrence in tumors that recurred or metastasized [8-10]. The recommended treatment of SFT is surgical resection. Despite this treatment, SFTs can recur and metastasize and have reported 10-year overall survival rates of 54-89% after a complete surgical resection [11].

However, surgical experience with SFTs of the liver is notably limited. In one series, 13.5% of 37 pleural SFTs metastasized [12] . SFT is rarely located in the liver parenchyma and usually follows a benign course. Few case reports in the literature describe a local recurrence of liver SFT and even fewer describe metastases [12-16]. In this study, we report a case of malignant liver SFT with skeletal metastasis and discuss the clinical presentation, previous diagnoses, radiological characteristics, histopathological and immunohistiochemical features, surgical treatment, adjunctive therapy and prognosis of SFTs with a review of the literature.

### Case report

**Figure 1:**
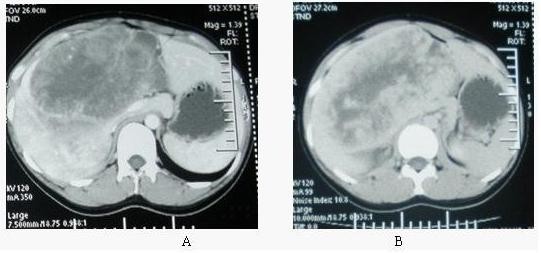
**Enhanced CT scan before hepatectomy**. A: Arterial phase image showing a large, heterogeneous mass of the liver with hypodense areas, which had clear border between tumor and the normal liver. Lesion showed heterogeneous enhancement and the high density of tumor blood vessels was seen in this region, lower density area to the central region as the center radial arrangement. Left hepatic vein was be compressed by the tumour and turned out to be rigidity. B: Venous late phase images showing a large, solid mass of the liver. Large hypodense tumor of the right hepatic lobe with an enhancement of the peripheral portion. The tumour was diffuse enhanced with a lager hypodense area in the center thought to be necrosis.

A 24-year-old, unmarried, nulliparous woman had presented one month earlier with upper right abdominal discomfort, distention, and vague right quadrant pain. The patient was a shop assistant for 4 years with no known history of potential environmental hazardous substances exposure. In addition, she was also a nonsmoker. Except for being a carrier of hepatitis B, she had no relevant medical or family history. For the further evaluation of her symptoms, she was referred to the referral hospital, where a contrast enhanced computer tomography (CT) study revealed a 30-cm exophytic heterogeneous mass in the right lobe of the liver (Figure 1). In a 2-D view, the lesion appeared as a hypodense area without calcifications measuring 28 cm×18 cm. An arterial-phase infusion of contrast showed peripheral enhancements with thin linear areas of enhancement (probably vessel-related) in the lesion. In the portal phase, there was a dishomogeneous enhancement with multiple hypodense areas. In the late phase, the lesion showed a light enhancement (similar to normal liver tissue) with multiple strong hypodense areas. There was no presence of ascites, and the patient had been given hepatinica. The patient was admitted to our hospital for surgery.

**Figure 2:**
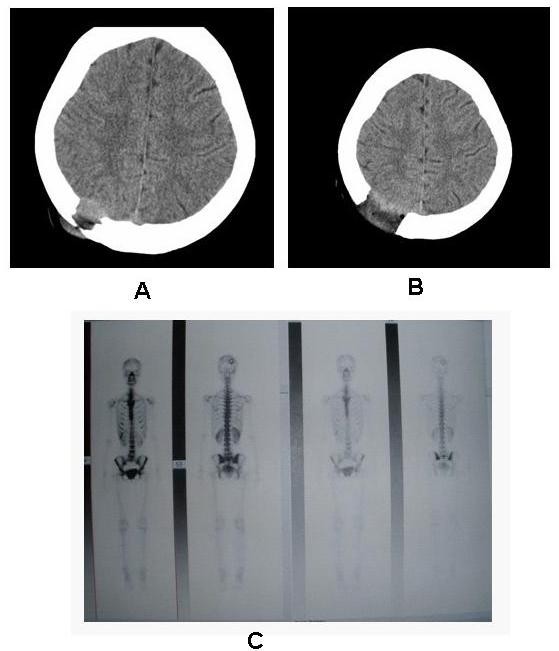
**Metastatic tumor of the right parietal bone**. A: CT scan of the brain shows that the right parietal bone is ruined before the craniectomy. B: CT scan of the brain after craniectomy. C: The emission-computed tomography scan shows an abnormal distribution of radioactivity in the right parietal bone area; the sparse distribution of radioactivity was central; peripheral distribution of radioactivity uptake before craniectomy.

On examination, abdominal distension was present. Palpation revealed tenderness and a large, firm mass in the right hypochondrium and epigastrium, and the liver was 12 cm below the lower edge of the ribs. There was no evidence of ascites, edema, hepatic failure, or cirrhosis stigmata. A firm mass of 4 cm×5 cm×3 cm was present on the right parietal bone without any tenderness (Figure 2). Laboratory tests, including routine biochemistry, liver function tests, urinalysis were normal, and tumor markers, such as serum carbohydrate antigen (CA)-125 was abnormal, whereas cancer antigen (CEA), α-fetoprotein, and CA19-9 levels were in the normal ranges. The patient's cranial CT scan revealed a parietal mass that was thought to be a metastatic tumor. On a whole-body skeletal scan, increased activity was found in the right parietal bone.

**Figure 3:**
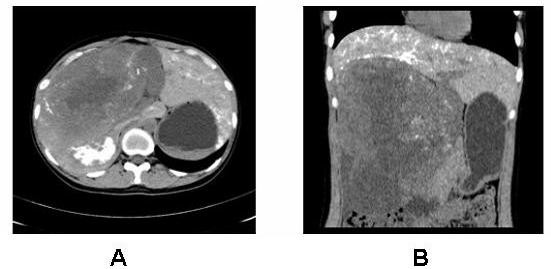
**CT scan after the treatment of TACE shows that there is almost no lipiodol deposits in the tumor area**.

**Figure 4:**
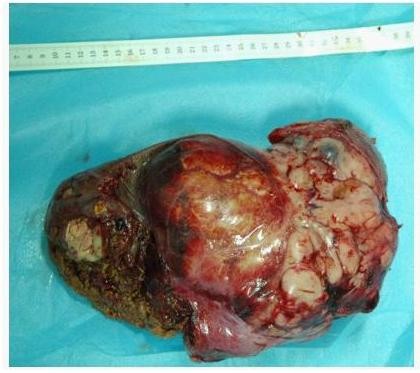
**The resected specimen, which measured 30 × 17 × 15 cm after an extended right hepatectomy**.

**Figure 5:**
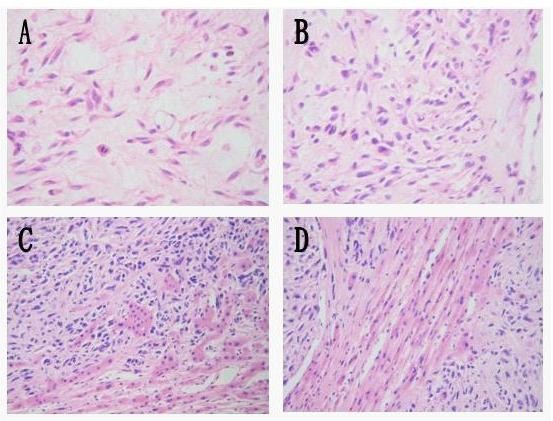
**Microscopic appearance**. (A) The mitosis of the nucleus. (B) Heteromorphism of the tumor. (C) and (D) Juncture of the tumor.

**Figure 6:**
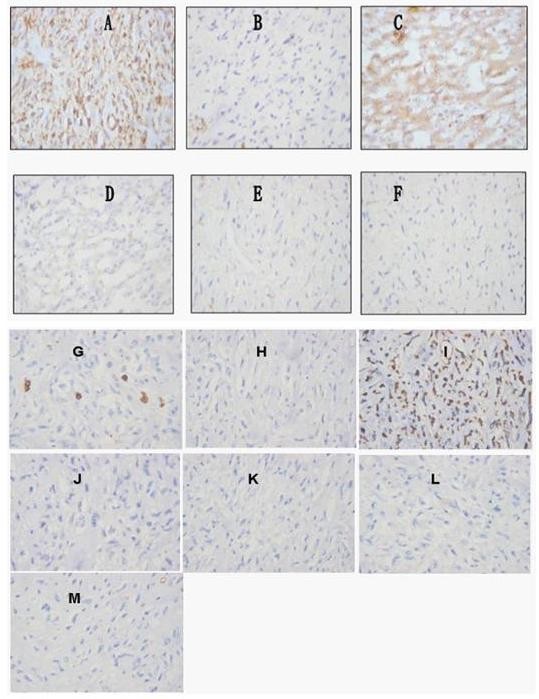
**Immunohistochemical features**. (A) Diffuse and strong CD34 immunopositivity. (B) Negative CD68 antigen staining. (C) Positive bcl-2 antigen staining. (D) Negative CD99 antigen staining. (E). Diffusely negative S-100 staining. (F) Negative smooth-muscle actin.(G) 3-5% positive Ki-67 antigen staining. (H) Negative cytokeratin antigen staining. (I) Positive vimentin antigen staining. (J) Negative synaptophysin antigen staining. (K) Negative calretinin. (L) Negative Desmin antigen staining. (M) Negative CD31 antigen staining.

The patient underwent angiography and transcatheter arterial chemoembolization (TACE, using diamminedichloroplatinum 60 mg, 5-fluorodeoxyuridine 1000 mg, Epirubicin 30 mg + 18-ml Lipiodol emulsifier) of the tumor-supplying vessels a few days before operation (Figure 3). A laparotomy was performed 8 days after admission and revealed that the tumor had pushed the colon to left and had adhered to the colon, stomach and omenta. An extended right hepatectomy was performed, and the postoperative course was uneventful. The gross examination revealed a large tumor measuring 30 × 17 × 15 cm and weighing 3750 g that was a firm, whitish, fibrotic nodule-encapsulated mass with necrosis (Figure 4). The resection margins were tumor-free. The tumor was located in the liver parenchyma, not in relation to the serosal surface. The cells were spindle-shaped tumor cells that were highly cellular and pleomorphic with areas of necrosis and a high mitotic index (> 10/hpf), haphazardly arranged in the classic short-storiform or so-called patternless pattern, and invading the surrounding liver tissue (Figure 5). The immunohistochemical analysis was strong positive for CD34, Bcl-2 and Vimentin. Ki-67 antigen staining was 3-5% positive. The tumor cells were negative for other markers, such as CD117, SM-actin, S-100, CD68, Myo-D1, CD99, Desmin, CD31, Calretinin, Synaptophysin, Cytokeratin, and Keratin (Figure 6). A craniectomy was performed 13 days after the extended right hepatectomy. The pathological examination showed skull base metastases from a malignant solitary fibrous tumor of the liver.

**Figure 7:**
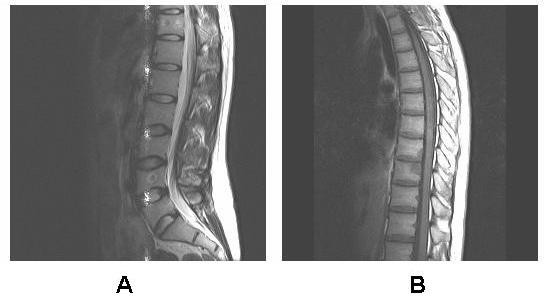
**Magnetic resonance imaging shows metastatic osteoblastic lesions of the thoracic and lumbar vertebrae 3 months after operation**.

A postoperative MRI examination in July 2010 demonstrated that the postsurgical residual lesion was still large. At follow-up one month later, the patient had severe back and waist pain. An MRI identified metastatic osteoblastic lesions of the thoracic and lumbar vertebrae (Figure 7).

The patient underwent four percutaneous ethanol-injection therapies (PEIs) and was treated with four rounds of chemotherapy that included adriamycin (40 mg on days 1-3), ifosfamide (3.0 g on days 1-4), and mesna (0.6 g IV 0 h, 4 h, and 8 h after IFO). Adjunctive radiation therapy was not administered. Despite the treatment, the tumor relapsed rapidly and was markedly enlarged. At follow-up seven months later, the patient was paralyzed; the patient died 16 months after the initial operation.

## Discussion

Hepatic SFTs are unusual neoplasms, with 38 previous cases worldwide being reported in the literature. SFTs typically affect adults aged 54.6 years (16-83) with no underlying predisposing factors. In a review article of 14 published cases, a strong female predilection was noted, and the average follow-up period was 27 months [17,18]. Most of these cases showed benign clinical characteristics, and only 3 cases had malignant variants. The clinicopathological features of the malignant hepatic SFT cases reported are summarized in Table 1. Three well-described cases, including the present case, came from one woman and two men with SFTs in the right lobe of the liver, and these patients ranged in age from 25 to 70 years (mean: 49). Immunohistochemically, the tumor cells showed strong vimentin immunostaining in all three cases and were positive for CD34 in two cases but negative for EMA and S-100 protein. The present case seems to be the first case of a hepatic malignant solitary fibrous tumor with skeletal metastases.

**Table 1:** Clinicopathological features of the reported malignant cases of liver solitary fibrous tumors

No	Reference	Year	Age/sex	Treatment	Size (cm)	Lobe of liver	Immunohistochemistry	Histology	Follow-up
1	Sezal Y, et al. [12]	2000	25/F	Excision	32 × 30 × 12	Right	Vimentin+	spindle cells with oval-fusiform nuclei	6 months
2	Chan G, et al. [13]	2007	70/M	Excision	27 × 24 × 12	Right	Vimentin+, CD34+, Bcl2+, CD99+	spindle-cell fibrosarcoma: highly cellular and pleomorphic, with areas of necrosis and a very high mitotic index (> 20/hpf)	> 9 months
3	Brochard C, et al. [14]	2010	54/M	Excision	17 × 12 × 8	Right	Vimentin+, CD34+, desmin+, actin+	Tumor cells were polymorphic: small and round, spindle-shaped, voluminous with acidophilic cytoplasm, or with polymorphous nuclei.	9 years

Clinically, hepatic SFTs cause symptoms only after reaching a certain size, or when vital structures are involved. SFTs are not known to grow invasively, and the course of the disease following complete surgical resection is usually benign. The patient may be asymptomatic or have non-specific clinical manifestations, including increased abdominal volume and circumference and abdominal pain, distention, and discomfort, as seen in our patient, or other symptoms, such as anorexia, nausea, vomiting and weight loss. Fever, hypoglycemia, abnormal liver tests and biliary-duct compression that leads to cholestasis and progressive jaundice are less commonly seen [12,13,16,19]. Laboratory results are usually normal; however, a few patients have had deranged liver function tests or elevated serum levels of a-fetoprotein [15,19,20]. Our patient had a high level of serum CA125.

The radiological findings of SFTs are unspecific and cannot distinguish between benign and malignant tumors. The diagnosis should be suspected if imaging reveals a solitary, highly vascular, well circumscribed, encapsulated mass that shows heterogeneous enhancement (due to the varied density of the collagen component) in CT and MRI images, especially if the enhancement is hypointense on the T2-weighted MRI and shows progressive enhancement in the delayed phase [20]. Most benign or malignant liver tumors, including hemangiomas, hepatic adenomas, focal nodular hyperplasia, hepatocellular carcinomas, fibrolamellar hepatocellular carcinomas and peripheral mass-forming cholangiocarcinomas, are predominantly hyperintense on the T2-weighted images, and some have a delayed, persistent enhancement [21]. The imaging features of benign and malignant solitary fibrous liver tumors appear to overlap. DWI can differentiate solitary fibrous tumors from malignant liver tumors. A whole-body fluorodeoxyglucose positron emission tomography (PET) showed that the mass had low metabolic activity and was probably benign [13,22].

Several authors believe that fine-needle aspiration also helps to establish a diagnosis, if the tissue sample is adequate and the fine-needle aspiration can be analyzed for immunomarkers; there has been one successful case report of this practice [23]. Other authors regard fine-needle biopsy to be inaccurate and do not recommend this practice. Large solid lesions of the liver (irrespective of the diagnosis), if deemed resectable at the pre- and intraoperative assessment, should be excised and sent for histological examination. Specimens obtained from a fine-needle biopsy only represent a small area of the tumor and may be misleading when entrapped proliferating bile ducts are mistaken for an adenocarcinoma, as in the present case. Alternatively, malignant foci may be missed, which would give a falsely reassuring result. Moreover, if the tumor is malignant, a percutaneous biopsy may seed tumor cells along the biopsy tract [7,22].

The tumor may be found on either the right or left side of the liver. The average tumor weight, where available, was 3,184 g and ranged from 1,850 g to 4,500 g. The mean of the largest diameter was 17.8 cm (2-32 cm)[18]. Paula Novais [24] presented a case of a large solitary hepatic fibrous tumor that was unresectable at first. Two years later, another attempt at surgical resction was made, but a life-threatening surgical complication occurred during the postoperative course. Unresectable, rare, non-hepatocellular or bile duct tumors that arise within the hepatic parenchyma are a formal indication for liver transplantation; therefore, liver transplantation can be considered for non-resectable SFTs. Because the patient was stable for 4 years, the author questioned such aggressive treatments in asymptomatic patients, due to the natural history of this tumor, which is not well known, and the risk of other complications.

In the present case, the liver SFT diagnosis was based on the association of characteristic histological and immunohistochemical features (i.e., high cellular proliferation of spindle cells arranged in a storiform pattern) with the immunohistochemical staining profile of CD34 (+), Bcl-2 (+) and SM-actin (-). These characteristics differentiate SFTs from other liver tumors, such as primary hepatocellular carcinomas (CD34-negative), leiomyomas (smooth-muscle actin-positive and CD34-negative) and mesotheliomas (vimentin-positive, CD34-positive, cytokeratin-positive) [25]. However, the CD34 antibody is not specific for SFT diagnosis and can be positive in angiosarcomas and gastrointestinal stromal tumors.

A hepatic SFT is often a benign neoplasm that has a good prognosis after surgery without recurrence. Most authors believe that postoperative adjuvant chemotherapy or radiotherapy should be reserved for incomplete resections and/or pathological features of malignancy [25,26]. Currently, the combination of doxorubicin and ifosfamide is the standard systemic chemotherapy regimen for many subtypes of soft-tissue sarcomas. Gemcitabine with docetaxel has also emerged as a good therapeutic choice for these patients. Although cases of SFT that respond to these chemotherapeutic agents have been reported sporadically, no systematic review or clinical trial has identified an effective systemic regimen for an unresectable SFT to date[11]. However, it has been proven that standard chemotherapy regimens may only have limited efficacy in SFT. The combination of temozolomide and bevacizumab had a remarkably high rate of overall response and a favorable duration of disease control; temozolomide/bevacizumab is a promising therapeutic regimen that warrants further investigation.

When complete resection is impossible or when there is recurrence, radiotherapy can be used, but a negative surgical margin does not mean that radiotherapy is unnecessary. Postoperative radiotherapy may theoretically reduce the risk of local recurrence; therefore, postoperative radiotherapy should be used as an adjunctive therapy in all patients [27]. In our case, we also used TACE for arterial embolization several days before operation. TACE is an option to generate tumor hypotrophy for a better resection and reduced hemorrhagic risk. Almost all reports agree that a complete surgical resection with tumor-free margins remains the cornerstone of treatment because of the reported 10-year overall survival rates of 54-89% [11]. The current case had widespread skeletal metastases; therefore, we used chemotherapy (adriamycin and ifosfamide), but this systemic regimen seemed to be inadequate. We believe that this report will contribute to understanding the natural history of liver SFTs.

SFTs of the liver are a rare neoplasm that cannot be definitely diagnosed by characteristic histological and immunohistochemical properties. A complete surgical resection with tumor-free margins is the best choice for treatment. Postoperative adjuvant chemotherapy or radiotherapy should be used in malignant SFTs; however, the use of these therapies in benign SFTs is still controversial.

### Consent

Written informed consent was obtained from the patient for publication of this case report and accompanying images. A copy of the written consent is available for review by the Editor-in-Chief of this journal.

## Competing interests

The authors declare that they have no competing interests.

## Authors' contributions

QL participated in the design of the study, and drafted the final version of the manuscript. LP participated in design of the study and manuscript drafting, carried out histopathological evaluation. YL participated in histopathological evaluation and helped in drafting the manuscript. YA tracked the clinical data. ZL helped in drafting the manuscript. YH tracked the clinical data. All authors read and approved the final manuscript.
